# Closure of Dedicated TAVR Device for Aortic Regurgitation in LVAD Patients: An Expected Consequence?

**DOI:** 10.1016/j.jaccas.2026.107151

**Published:** 2026-03-04

**Authors:** Sara Bombace, Karl-Philip Rommel, Mehmet Oezkur, Philipp Lurz, Ralph Stephan von Bardeleben, Martin Geyer

**Affiliations:** a Department of Cardiology, University Medical Center of the Johannes Gutenberg-University Mainz, Mainz, Germany; b Department of Cardiac and Vascular Surgery, University Medical Center of the Johannes Gutenberg-University Mainz, Mainz, Germany

**Keywords:** aortic regurgitation, left ventricular assist device, transcatheter aortic valve replacement, valve thrombosis

## Abstract

Severe aortic regurgitation (AR) in patients with left ventricular assist device (LVAD) causes recirculatory flow and hemodynamic compromise. Two LVAD patients with severe AR underwent transfemoral JenaValve Trilogy implantation. Both procedures achieved immediate abolition of regurgitation; however, both prostheses subsequently showed valve closure, with one case progressing to complete thrombotic sealing. Despite closed prosthetic valves, both patients demonstrated improved LVAD efficiency and absence of clinical embolic events at last available follow-up. JenaValve Trilogy implantation can provide hemodynamic benefit in high-risk LVAD patients, even when functional or thrombotic valve closure occurs. However, long-term durability and anticoagulation strategies remain to be determined.

## Visual Summary

**Figure:**
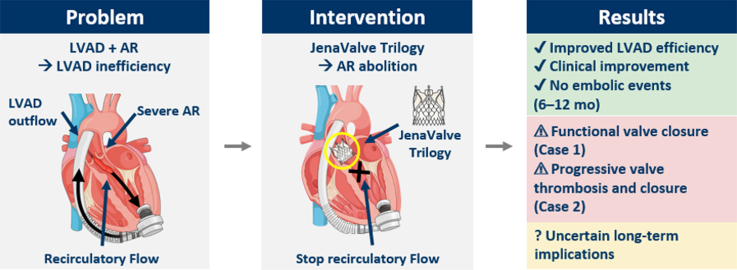

Left ventricular assist devices (LVADs) provide durable circulatory support for patients with advanced heart failure, but they are associated with specific long-term complications, including the development of aortic regurgitation (AR). The pathophysiology of LVAD-associated AR is multifactorial. Continuous-flow LVAD support creates persistently elevated transvalvular pressure gradients and reduced aortic valve opening, leading to leaflet stasis, commissural fusion, and progressive valve degeneration, further promoted by abnormal shear stress within the ascending aorta.^1^^,^^2^ Additional risk factors for AR during LVAD support, beyond a persistently closed aortic valve, include advanced age, female sex, lower body surface area, systemic hypertension, enlarged aortic root diameter, and longer duration of LVAD support.^1^^,^^2^

**Take-Home Messages**

• JenaValve Trilogy TAVR can provide hemodynamic benefit in LVAD patients with severe AR through complete AR abolition, even when the prosthetic valve remains closed.
• Preprocedural native leaflet motion does not seem to predict post-TAVR valve function.
• Valve thrombosis can occur despite therapeutic anticoagulation, necessitating informed consent regarding long-term risks as well as close echocardiographic surveillance in this high-risk population.

Progressive AR may result in a deleterious recirculatory loop, reducing effective LVAD forward flow and ultimately precipitating heart failure decompensation. Surgical correction of AR in LVAD patients often carries prohibitive risk. Transcatheter aortic valve replacement (TAVR) has therefore emerged as a potentially feasible alternative. In this report, we describe 2 LVAD patients with severe AR treated with transfemoral implantation of the JenaValve Trilogy system, highlighting hemodynamic effects and device-related considerations.

## Case Presentation

### Case 1

A 67-year-old woman with dilated cardiomyopathy and LVAD implanted 2 years earlier presented with acute cardiac decompensation. Her medical history included percutaneous mitral and tricuspid valve repair, recurrent driveline infection, chronic kidney disease, and implantable cardioverter-defibrillator placement. On admission, she was in cardiogenic shock despite maximal inotropic support, with acute on chronic renal impairment requiring temporary dialysis and hepatic dysfunction. She had been declined for urgent heart transplantation.

### Case 2

A 65-year-old man with terminal ischemic cardiomyopathy and LVAD implanted 7 years earlier was admitted with progressive heart failure symptoms with NYHA functional class III/IV status. His medical history included 3-vessel coronary artery disease treated with percutaneous and surgical revascularization, previous left ventricular apical thrombus, and implantable cardioverter-defibrillator placement. He had been listed for heart transplantation for over 5 years.

Both patients were on therapeutic anticoagulation with vitamin K antagonist (target international normalized ratio: 2-3).

## Diagnostic Work-Up

### Case 1

**Figure 1:**
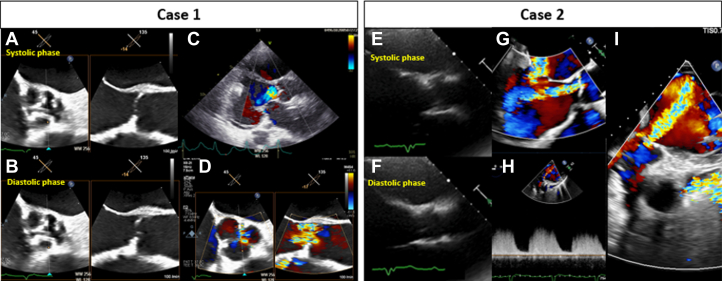
Baseline Echocardiographic Images for Cases 1 and 2 Case 1: (A and B) The aortic valve is shown in systole and diastole, respectively, demonstrating absent leaflet motion with a central coaptation defect. (C and D) Severe central aortic regurgitation is demonstrated on color Doppler imaging. Case 2: (E and F) The aortic valve is shown in systole and diastole, respectively, demonstrating preserved leaflet motion. (G and I) severe central aortic regurgitation is demonstrated on color Doppler imaging. (H) Continuous-wave Doppler demonstrates regurgitant flow extending beyond diastole and occupying a substantial portion of the cardiac cycle.

Transthoracic echocardiography (TTE) and transesophageal echocardiography (TEE) demonstrated an extremely reduced left ventricular ejection fraction of 5%. The native aortic leaflets showed absent systolic opening, were calcified, and exhibited a central coaptation gap, resulting in severe central AR with continuous regurgitant flow throughout the cardiac cycle and a vena contracta width of 11.0 mm. Despite absent leaflet motion, no evidence of aortic valve thrombosis was observed (Figures 1A to 1D, Videos 1 and 2). Surgical risk was prohibitive (EuroSCORE II: 36.19%). These findings established the diagnosis of severe AR, causing cardiogenic shock and multiorgan failure.

### Case 2

TTE and TEE showed a severely reduced left ventricular ejection fraction of 25%. Severe central AR was present, with a vena contracta width of 5.8 mm and regurgitant flow extending beyond diastole on continuous-wave Doppler imaging (Figures 1E to 1I). Surgical aortic valve replacement carried prohibitive risk (EuroSCORE II: 35.99%). These findings confirmed severe AR leading to LVAD recirculation and progressive heart failure decompensation.

In both cases, the heart team determined that AR elimination was necessary to interrupt the LVAD-AR cycle. Given prohibitive surgical risk and absence of transplant options, transfemoral TAVR was selected as definitive therapy.

## Management

Both procedures were performed transfemorally using the JenaValve Trilogy, which clips to native leaflets using 3 locators, providing stable fixation even in patients with minimal or no calcification.^3^ Valve sizing was performed according to standard practice, based on preprocedural computed tomography and validated manufacturer sizing charts (Supplemental Figures 1 and 2). No changes to LVAD settings were required before or during TAVR deployment in either case. LVAD parameters before and after TAVR are provided in Supplemental Tables 1 and 2. Cerebral embolic protection was omitted given the absence of thrombotic or valvular high-risk features. Rapid ventricular pacing was not required for valve deployment in the setting of continuous LVAD flow. In both cases, valve crossing was feasible using standard techniques.

### Case 1

**Figure 2:**
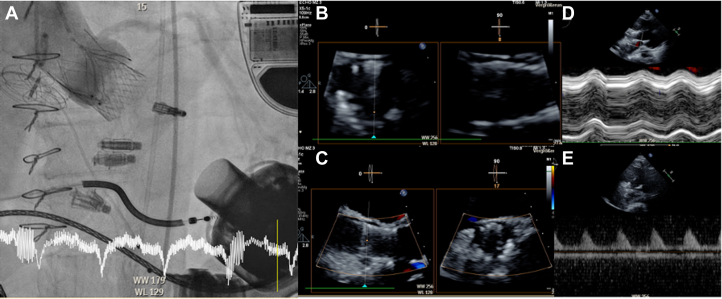
Case 1: Postimplantation Imaging (A) Postimplantation aortography confirms correct valve positioning. (B-E) Multimodal echocardiographic assessment including (C) color Doppler, (D) M-mode, and (E) continuous-wave Doppler all demonstrate absent transvalvular flow, consistent with hemodynamic valve closure due to insufficient opening gradient.

**Figure 3:**
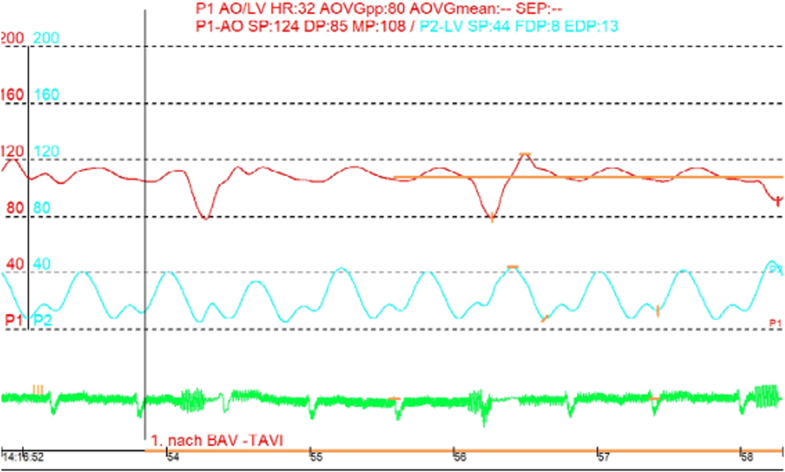
Case 1: Invasive Hemodynamic Pressure Tracings Immediately After JenaValve Trilogy Implantation The red tracing represents aortic pressure, and the blue tracing represents left ventricular pressure. Left ventricular pressure remains consistently below aortic pressure throughout the cardiac cycle, with complete absence of a transvalvular gradient, rendering valve opening hemodynamically impossible.

A 23-mm JenaValve Trilogy was deployed successfully, with immediate elimination of AR (Figure 2). Postimplantation invasive pressure tracings revealed consistently low left ventricular pressure that never exceeded aortic pressure, with complete absence of transvalvular gradient, rendering valve opening hemodynamically impossible (Figure 3).

Postprocedural imaging revealed no leaflet motion and absent transvalvular flow on color, M-mode, and continuous-wave Doppler, consistent with functional valve closure (Figures 2B to 2E). Despite this, the patient improved clinically and was weaned from inotropes.

### Case 2

**Figure 4:**
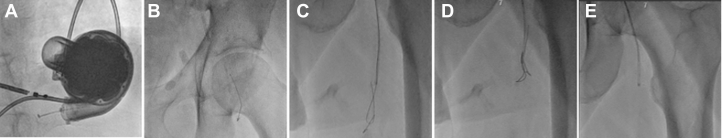
Case 2: Intraoperative Fluoroscopy (A) Intraoperative fluoroscopy showing embolized J-wire fragment visible in the LVAD outflow graft, and (B-E) successful interventional retrieval of the wire fragment using a crossover technique with a snare device via contralateral femoral access. LVAD = left ventricular assist device.

**Figure 5:**
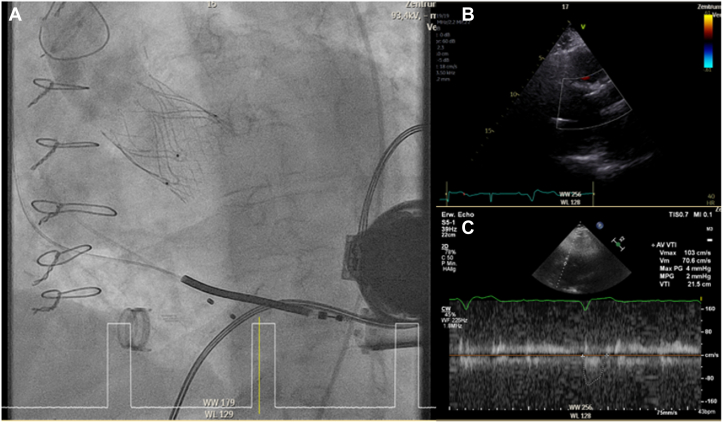
Case 2: Postimplantation Imaging (A) Postimplantation aortography. (B and C) Transthoracic echocardiography postprocedure with poorly visualized transvalvular flow on color Doppler and minimal transvalvular gradients.

**Figure 6:**
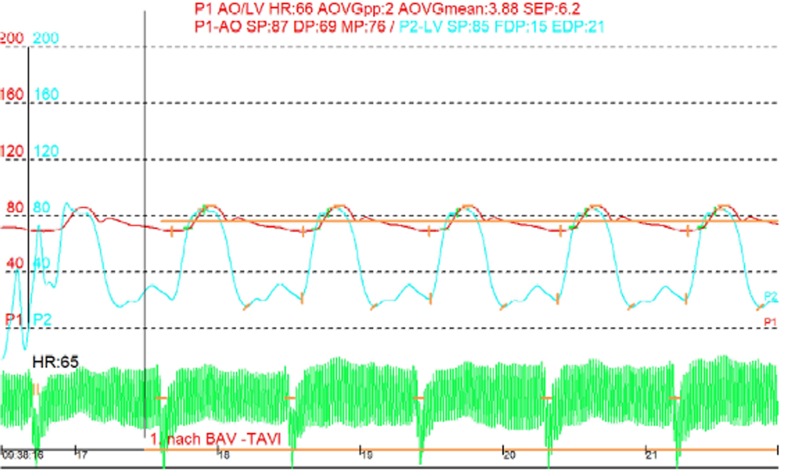
Case 2: Invasive Hemodynamic Pressure Tracings Immediately After JenaValve Trilogy Implantation The red tracing represents aortic pressure, and the blue tracing represents left ventricular pressure. Left ventricular pressure intermittently exceeds aortic pressure during systole, creating brief periods where a transvalvular gradient exists (mean gradient: 3.88 mm Hg), theoretically permitting intermittent leaflet opening.

A 27-mm JenaValve Trilogy was implanted successfully with optimal positioning and complete abolition of AR. A guidewire (J-wire) fracture and peripheral embolization of the fragment through the LVAD occurred intraprocedurally without compromising LVAD function; the fragment was able to be retrieved percutaneously via crossover snare without sequelae (Figures 4 and 5). Postimplantation invasive pressure tracings showed left ventricular pressure intermittently exceeding aortic pressure with a minimal transvalvular gradient, theoretically permitting intermittent leaflet opening (Figure 6). Postprocedural echocardiography showed poor visualization of leaflet motion and transvalvular flow (Figures 5B and 5C).

In both cases, LVAD flow and power consumption decreased after TAVR, consistent with elimination of the recirculatory AR loop and resulting in more efficient LVAD function.

## Follow-Up and Outcome

### Case 1

The patient remained clinically stable after discharge over the subsequent 6 months, with preserved LVAD function and no clinical evidence of thromboembolic events.

### Case 2

**Figure 7:**
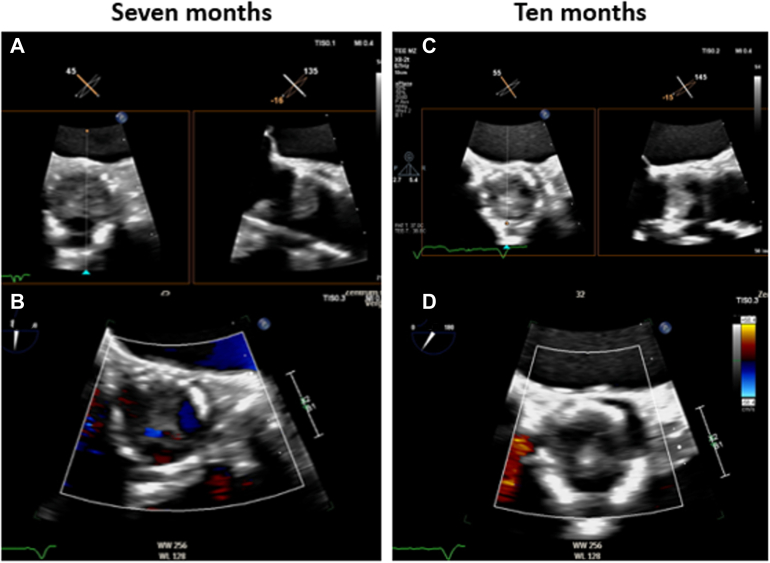
Case 2: Transesophageal Echocardiography at 7- and 10-Month Follow-Up (A and B) TEE at the 7-month follow-up during investigation for bacteremia, demonstrating subtotal valve thrombosis with minimal residual central forward flow. (C and D) TEE at the 10-month follow-up confirming complete thrombotic sealing with no transvalvular flow. TEE = transesophageal echocardiography.

**Figure 8:**
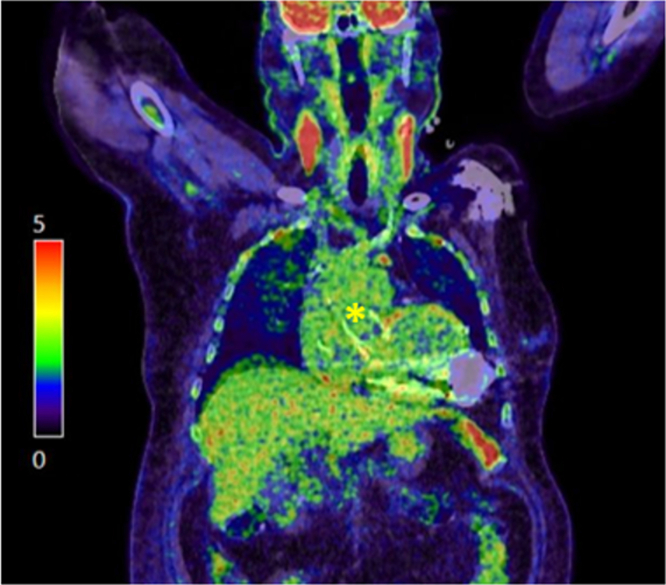
Case 2: PET/CT Fusion Image Showing Biodistribution of the Radiotracer (^18^F-Fluorodeoxyglucose) No focal pathological uptake was identified at the site of the transcatheter aortic valve prosthesis, particularly at the leaflet level (yellow asterisk), excluding endocarditis. PET/CT = positron emission tomography/computed tomography.

Seven months postprocedure, the patient developed *Staphylococcus* *aureus* bacteremia. TEE revealed subtotal thrombosis of the transcatheter valve with minimal residual flow without vegetations (Figures 7A and 7B; Videos 3 and 4). Positron emission tomography/computed tomography excluded endocarditis (Figure 8). The patient received guideline-directed antimicrobial therapy with documented clearance of blood cultures. A TEE performed 10 months postprocedure demonstrated complete thrombotic valve sealing with no residual flow (Figures 7C and 7D; Videos 5 and 6). At the latest available follow-up (12 months), the patient remained stable (NYHA functional class II-III) under standard LVAD anticoagulation without thromboembolic events.

## Discussion

This report describes 2 LVAD patients with severe AR who underwent successful JenaValve Trilogy implantation, achieving complete AR abolition and clinical stabilization. However, both prostheses developed valve closure, raising important questions about device performance in the unique LVAD hemodynamic environment.

Importantly, assessment of AR severity in LVAD patients is inherently challenging using echocardiography, as continuous retrograde LVAD flow alters regurgitant timing and renders conventional quantitative parameters less reliable. Regurgitant flow may extend beyond diastole and persist throughout the cardiac cycle, leading to substantial volume overload and LVAD recirculation despite only moderate values of traditional geometric indices.^4^

Our findings align with emerging data on TAVR in LVAD patients. A recent study demonstrated the feasibility and safety of JenaValve Trilogy implantation in 17 LVAD patients, reporting 88.2% device success and no migration, though valve closure and thrombosis were not addressed.^5^ Another study analyzed 27 LVAD patients treated with various TAVR devices. Among them, only 4 (14.8%) received the JenaValve Trilogy. At the 12-month follow-up, 80.8% of all patients exhibited valve closure and 31.8% had subclinical thrombosis, but the JenaValve Trilogy subgroup was not separately analyzed.^6^ These data suggest that valve closure may represent a predictable consequence of altered hemodynamics rather than an isolated complication.

The mechanisms underlying valve closure in our cases likely differ. In case 1, functional closure resulted from hemodynamically insufficient transvalvular gradients immediately postimplantation, as evidenced by invasive pressure tracings showing left ventricular pressure consistently below aortic pressure. In case 2, despite initial hemodynamic conditions theoretically permitting intermittent leaflet opening, progressive thrombotic sealing occurred over 10 months. Whether the *S* *aureus* bacteremia at 7 months contributed to thrombus formation remains speculative, though temporal association is notable.

Preprocedural native leaflet motion did not predict post-TAVR valve function, as case 2 demonstrated preserved baseline leaflet opening yet developed complete thrombotic closure. Additionally, therapeutic anticoagulation (international normalized ratio: 2-3) failed to prevent thrombosis in case 2, raising the question of whether intensified antithrombotic strategies might mitigate this risk. Case 2 also illustrates the procedural challenge of guidewire manipulation near the LVAD inflow cannula, emphasizing the need for meticulous attention to avoid guidewire complications.

Despite valve closure, both patients demonstrated hemodynamic benefit without embolic events during available, albeit limited, follow-up, suggesting that AR abolition, even with a closed prosthesis, interrupts the detrimental LVAD-AR recirculatory flow.

## Conclusions

In LVAD patients with severe AR who are ineligible for surgery or transplantation, the JenaValve Trilogy achieves complete AR abolition, even when resulting in valve closure. This closure may be common yet does not preclude hemodynamic benefit. However, functional valve closure may predispose to thrombotic or infectious complications, which must be weighed against the substantial hemodynamic benefit. Key questions regarding long-term durability, optimal anticoagulation strategy, and the clinical implications of valve closure remain unresolved. Until further data emerge, TAVR in LVAD patients should be individualized, balancing short-term hemodynamic gains against uncertain long-term risks.

**Visual Summary:**
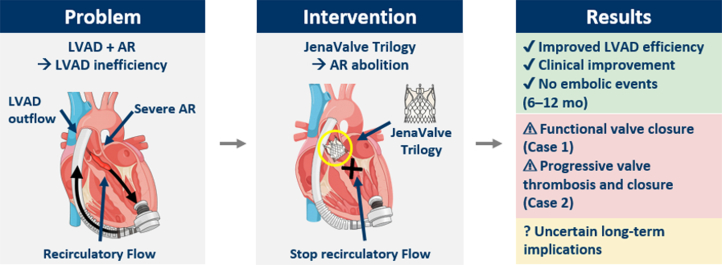
TAVR With a Dedicated Device for Aortic Regurgitation in LVAD Patients: Hemodynamic Benefit Despite Aortic Valve Closure (Left) Severe AR in patients with LVAD creates recirculatory flow that compromises LVAD efficiency. (Middle) Transfemoral JenaValve Trilogy implantation eliminates AR and interrupts this detrimental cycle. (Right) Both patients achieved improved LVAD performance and clinical stabilization without embolic events during follow-up. However, device-related complications occurred: functional valve closure without thrombosis in case 1, and progressive thrombotic valve sealing in case 2. The long-term implications of prosthetic valve closure in this unique hemodynamic setting remain uncertain. AR = aortic regurgitation; LVAD = left ventricular assist device.

## Funding Support and Author Disclosures

Dr Geyer has served as a proctor for JenaValve Technology. All other authors have reported that they have no relationships relevant to the contents of this paper to disclose.

## Appendix

Video 1 Case 1 – Baseline TEE showing degenerative aortic valve with absent leaflet motion Case 1: Transesophageal Echocardiography Demonstrating Severe Central Aortic Regurgitation With No Native Leaflet Opening During Systole

Video 2 Case 1 – Baseline TEE demonstrating severe aortic regurgitation Case 1: Transesophageal Echocardiography Demonstrating Severe Central Aortic Regurgitation With No Native Leaflet Opening During Systole

Video 3 Case 2 – Subtotal valve thrombosis at 7-month follow-up Case 2: Transesophageal Echocardiography at 7 Months Showing Subtotal Valve Thrombosis With Minimal Residual Flow

Video 4 Case 2 – Minimal residual central flow at 7-month follow-up Case 2: Transesophageal Echocardiography at 7 Months Showing Subtotal Valve Thrombosis With Minimal Residual Flow

Video 5 Case 2 – Complete thrombotic valve sealing at 10-month follow-up Case 2: Transesophageal Echocardiography at 10 Months Demonstrating Complete Thrombotic Valve Sealing

Video 6 Case 2 – Absence of transvalvular flow at 10-month follow-up Case 2: Transesophageal Echocardiography at 10 Months Demonstrating Complete Thrombotic Valve Sealing
