# Supplementary material for: Closure of Dedicated TAVR Device for Aortic Regurgitation in LVAD Patients: An Expected Consequence?
Source: JACC Case Rep. 2026 Mar 4;31(14):107151. doi: 10.1016/j.jaccas.2026.107151 (PMC13080573; doi:10.1016/j.jaccas.2026.107151)
Supplement: Supplemental Material [file mmc7.docx]

Supplemental material

[Table S1. 2](#_Toc219745303)

[Table S2. 2](#_Toc219745304)

[Figure S1. 3](#_Toc219745305)

[Figure S2. 4](#_Toc219745306)

# Table S1. LVAD Parameters Before and After TAVR in Case 1

| **Case 1** | **Pre** | **Post** |
| --- | --- | --- |
| Speed (RPM) | 5800 | 5800 |
| Flow (LPM) | 5.5 | 5.0 |
| Pulsatility Index | 2.9 | 1.8 |
| Power (Watt) | 5.1 | 4.6 |

**Table S1.** Case 1 was supported with a HeartMate 3 device, which reports a Pulsatility Index. Abbreviations: LPM = liters per minute; LVAD = left ventricular assist device; RPM = revolutions per minute; TAVR = transcatheter aortic valve replacement.

# Table S2. LVAD Parameters Before and After TAVR in Case 2

| **Case 2** | **Pre** | **Post** |
| --- | --- | --- |
| Speed (RPM) | 3000 | 3000 |
| Flow (LPM) | 5.6 | 4.4 |
| Pulsatility (LPM) | 2.4 | 3.0 |
| Power (Watt) | 5.9 | 5.2 |

**Table S2.** Case 2 was supported with a Medtronic HVAD, in which pulsatility is expressed as Pulsatility (L/min). Abbreviations: LPM = liters per minute; LVAD = left ventricular assist device; RPM = revolutions per minute; TAVR = transcatheter aortic valve replacement.

# Figure S1.


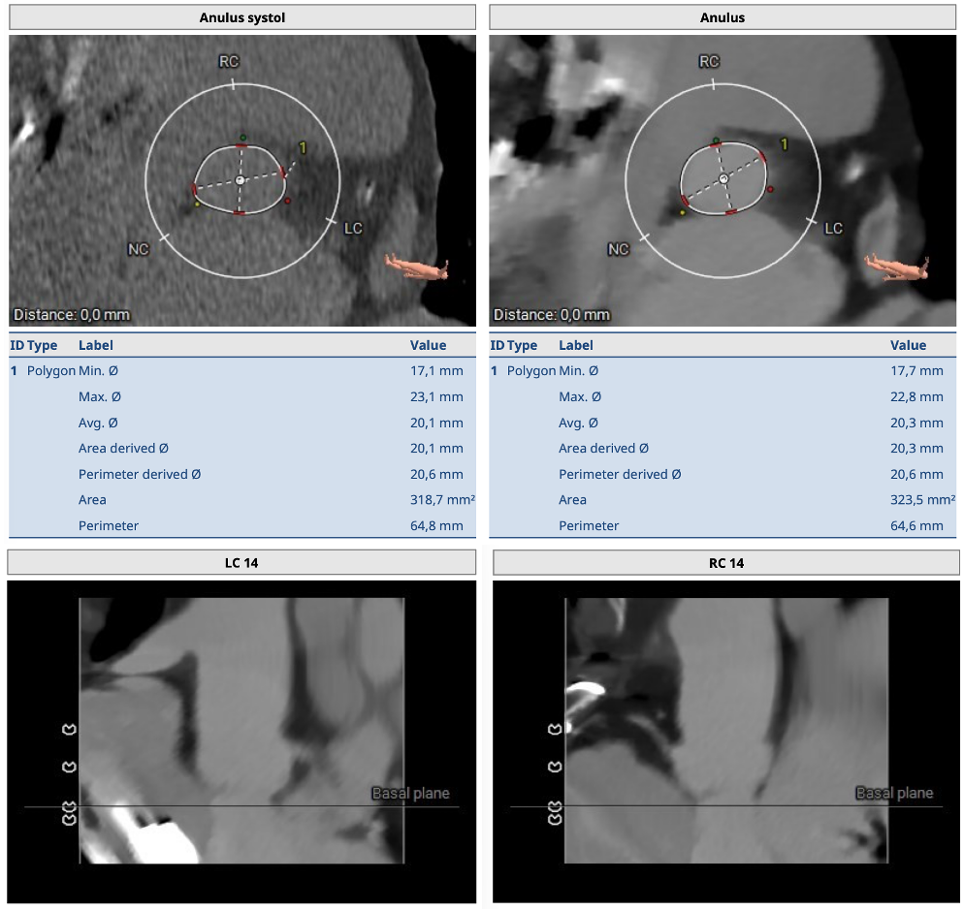


**Figure S1. Pre-procedural Computed Tomography Assessment in Case 1**

Computed tomography showing multiplanar reconstruction of the aortic annulus in systole (upper left) and diastole (upper right). Based on these annular measurements, a 23 mm JenaValve Trilogy prosthesis was selected. Coronary ostial height measurements are shown in the lower panels: left main coronary artery (bottom left, 14 mm) and right coronary artery (bottom right, 14 mm), measured perpendicular to the annular plane to assess the risk of coronary obstruction during transcatheter aortic valve replacement.

# Figure S2.


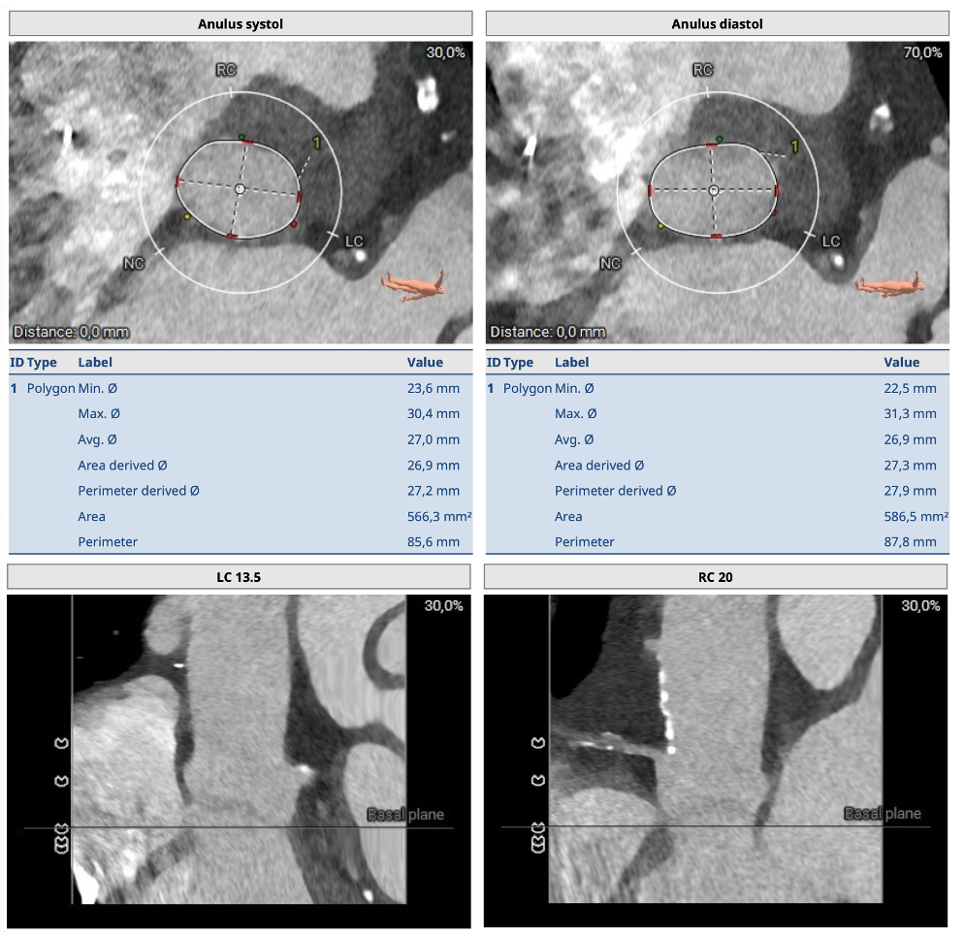
**Figure S2. Pre-procedural Computed Tomography Assessment in Case 2**

Computed tomography showing multiplanar reconstruction of the aortic annulus in systole (upper left) and diastole (upper right). Based on these annular measurements, a 27 mm JenaValve Trilogy prosthesis was selected. Coronary ostial height measurements are shown in the lower panels: left main coronary artery (bottom left, 13.5 mm) and right coronary artery (bottom right, 20 mm), measured perpendicular to the annular plane to assess the risk of coronary obstruction during transcatheter aortic valve replacement.
